# Psychometric validation of a multi-dimensional capability instrument for outcome measurement in mental health research (OxCAP-MH)

**DOI:** 10.1186/s12955-017-0825-3

**Published:** 2017-12-28

**Authors:** Francis Vergunst, Crispin Jenkinson, Tom Burns, Paul Anand, Alastair Gray, Jorun Rugkåsa, Judit Simon

**Affiliations:** 1 Research Unit on Children’s Psychosocial Maladjustment, University of Montréal, Ste Justine Hospital, 3175 Chemin de la Côte Ste-Catherine, Montréal, H3T 1C5 Canada; 2 Department of Psychiatry, University of Oxford, Warneford Hospital, Oxford, OX3 7JX UK; 3 Health Services Research Unit, Nuffield Department of Population Health, University of Oxford, Richard Doll Building, Old Road Campus, Oxford, OX3 7LF UK; 4 Faculty of Arts and Social Sciences, The Open University, Milton Keynes, MK7 6AA UK; 5 Centre for Philosophy of Natural and Social Sciences, London School of Economics, London, WC2A 2AE UK; 6 Department of Social Policy and Intervention, University of Oxford, 32 -37 Wellington Square, Oxford, OX1 2ER UK; 7 Health Economics Research Centre, Nuffield Department of Population Health, University of Oxford, Richard Doll Building, Old Road Campus, Oxford, OX3 7LF UK; 8 Health Services Research Unit, Akershus University Hospital, 1478 Lørenskog, Norway; 9 Centre for Care Research, University College of Southeast Norway, 3900 Porsgrunn, Norway; 10 Department of Health Economics, Center for Public Health, Medical University of Vienna, Kinderspitalgasse 15, 1090 Vienna, Austria

**Keywords:** Outcome measurement, Capabilities, Psychometric validation, Mental health, Psychiatry, Psychosis, Community treatment orders

## Abstract

**Background:**

Patient reported outcome measures (PROMs) are widely used in mental healthcare research for quality of life assessment but most fail to capture the breadth of health and non-health domains that can be impacted. We report the psychometric validation of a novel, multi-dimensional instrument based on Amartya Sen’s capability approach intended for use as an outcome measure in mental health research.

**Methods:**

The Oxford Capabilities Questionnaire for Mental Health (OxCAP-MH) is a 16-item self-complete capability measure that covers multiple domains of functioning and welfare. Data for validation of the instrument were collected through a national randomised controlled trial of community treatment orders for patients with psychosis. Complete OxCAP-MH data were available for 172 participants. Internal consistency was established with Cronbach’s alpha; an interclass correlation coefficient was used to assess test-retest reliability in a sub-sample (*N* = 50) tested one week apart. Construct validity was established by comparing OxCAP-MH total scores with established instruments of illness severity and functioning: EuroQol (EQ-5D), Brief Psychiatric Rating Scale (BPRS), Global Assessment of Functioning (GAF) and Objective Social Outcomes Index (SIX). Sensitivity was established by calculating standard error of measurement using distributional methods.

**Results:**

The OxCAP-MH showed good internal consistency (Cronbach’s alpha 0.79) and test-retest reliability (ICC = 0.86). Convergent validity was evidenced by strong correlations with the EQ-5D (VAS 0.52, *p* < .001) (Utility 0.45, *p* < .001), and divergent validity through more modest associations with the BPRS (−0.41, *p* < .001), GAF (0.24, *p* < .001) and SIX (0.12, *p* = ns). A change of 9.2 points on a 0–100 scale was found to be meaningful on statistical grounds.

**Conclusions:**

The OxCAP-MH has demonstrable reliability and construct validity and represents a promising multi-dimensional alternative to existing patient-reported outcome measures for quality of life used in mental health research.

**Electronic supplementary material:**

The online version of this article (10.1186/s12955-017-0825-3) contains supplementary material, which is available to authorized users.

## Background

Standardised outcome assessment is widely used in mental health care to evaluate health and social care interventions and to aid decisions about resource allocation. The use of patient reported outcome measures (PROMs) has become increasingly popular with the aim of improving measurement accuracy and increasing patient involvement and satisfaction [1]. The purpose of a PROM is to assess, from the patient’s perspective, the impact of illness or an intervention on their life [2]. Studies show that the systematic use of information generated from PROMs can improve health outcomes, enhance decision making and communication between doctors and patients and increase patient satisfaction with care [3–8]. Many established PROMs focus on a narrow range of outcomes, however, and consequently fail to capture the full range of health and non-health domains that can be impacted by an illness or intervention [9].

In the United Kingdom and Europe the EQ-5D is one of the most widely used generic health-related quality of life PROM [10–12]. The instrument is used in general and mental health contexts and endorsed by the UK National Institute for Health and Care Excellence (NICE) for the calculation of quality adjusted life years (QALY) used in cost-utility analyses [10, 11, 13]. However, the EQ-5D has been criticised on methodological and conceptual grounds. First, it focuses exclusively on health-related quality of life and consequently fails to capture non-health benefits and broader welfare inequalities, and, second, it has been shown to lack sensitivity when applied to mental health populations, particularly those with psychotic disorders and severe and complex non-psychotic disorders [9, 14].

Severe mental illnesses are complex health conditions that lead to poor outcomes in multiple life domains and there is a need for sophisticated, multi-dimensional PROMs that are able to capture the breadth of life domains that may be impacted. Amartya Sen’s [15–17] capability approach, which employs a rich set of dimensions for outcome evaluation, including health and non-health outcomes, has emerged as an important alternative conceptual framework for evaluating human wellbeing [12, 18–21].

According to Sen, well-being should be conceptualised in terms of a person’s ability to function – that is for the person to be and do the things that matter to them and that they have reason to value. These functionings can range from the rudimentary, such as being adequately nourished and housed to the more abstract, such as feeling socially valued or achieving self-respect. It is these ‘beings’ and ‘doings’, Sen argues, that make life valuable to the individual and worth living [16].

Sen distinguishes between functionings – what I do or am – and capabilities – what I am able or free to do or be. The distinction between achieved functionings and capabilities is between that which is realised and that which is effectively possible [22]. Sen illustrates this distinction with the example of a person who is starving and a person who is fasting. In both cases, the functional outcome is the same – they don’t eat – but their capabilities are different: the first person does not have the capability to eat while the second person does, but chooses not to exercise it. What matters for well-being, then, is not what functionings an individual has achieved, but rather the genuine opportunities to achieve the functionings that matter to that individual.

Health economists and social scientists increasingly agree that the capability approach offers a richer, more nuanced theoretical background to the evaluation of welfare, when compared with the traditional utilitarian welfarism of QALYs [12, 19, 23].

A small and growing number of capability measures have been developed for use in a variety of health contexts [18]. Within the capability literature the question of which capabilities are most relevant for individuals or groups within a given context remains the subject of much debate. Sen declined to provide an authoritative list of ‘essential’ capabilities necessary for the good life, arguing that such a list would necessarily vary, across time and place, and that individuals and communities are best placed to decide on such a list. Nussbaum [24] proposed a list of ten ‘essential’ capabilities including*: ‘life’, ‘bodily health’, ‘bodily integrity’, ‘senses, imagination and thought’, ‘emotions’, ‘practical reason’, ‘affiliation’, ‘other species’, ‘play’, and ‘control over one’s environment’*. Although Nussbaum’s list is the most widely accepted, several other lists have emerged, most of which are highly generic and contain considerable conceptual overlap with one another [25].

The OxCAP-MH is the first instrument developed and operationalised for use as an outcome measure in mental health research [26]. The instrument is designed to capture the substantive freedoms that an individual has to be and do the things that they have reason to value across multiple life domains including: *performing usual activities, meeting socially with friends, not losing sleep over worry, enjoying recreational activities, having suitable accommodation, feeling safe, freedom from discrimination, freedom from assault (including sexual and domestic), ability to influence local decisions, freedom to express personal views, appreciation of nature, respecting and valuing people, enjoying love friendship and support, self-determination, freedom of artistic expression* and *access to interesting activities*. A more thorough discussion of the development of the instrument as well as the theoretical background to the capability approach and its application in the mental health context is available elsewhere [26, 27].

Initial testing of OxCAP-MH 16-item index indicates both the feasibility and face validity of directly measuring capabilities in patients with severe mental illness [26]. However, further work is required to establish the instruments’ broader psychometric properties including internal consistency, test-retest reliability and convergent and discriminant validity and sensitivity to change.

### Construct validity

The construct validity of the OxCAP-MH was evaluated by comparing total scores of the OxCAP-MH to those of previously validated measures of health-related quality of life (EQ-5D), overall functioning (GAF), psychiatric symptoms (BPRS), and objective social outcomes (SIX). Convergent validity was assessed by examining the association between the OxCAP-MH and the EQ-5D, while discriminant validity was determined by the associations between the OxCAP-MH, GAF, BPRS and SIX.

The EQ-5D was used to assess convergent validity because, among the instruments used in this study, it was believed on theoretical grounds, to be the most closely related to the OxCAP-MH. It was hypothesised that the OxCAP-MH would correlate strongly with the EQ-5D since the latter captures health-related quality of life (which should overlap with the OxCAP-MH’s concept of wellbeing), and both are multi-dimensional, subjective, patient-reported outcome measures. In contrast, it was hypothesised that the association between the OxCAP-MH and the GAF and BPRS would be modest on the grounds that these instruments represent the clinician/researcher’s impression of the patient’s overall functioning and symptom severity respectively, while the association with the SIX would be quite weak, since this instrument captures only “objective facts” about the patient’s social situation, such as whether they have employment and housing. It was expected that OxCAP-MH would be positively correlated with the GAF and SIX and negatively correlated with the BPRS, since higher BPRS scores indicate greater symptom severity. These latter three measures are widely used within psychiatry whereas the EQ-5D is more commonly used in economics. Compared with all these, our measure explicitly monitors a wide set of aspects of quality of life.

### Sensitivity to change

Sensitivity to change refers to the ability of the instrument to measure any degree of change, while responsiveness reflects the ability to detect change over time that is clinically meaningful [28]. Sensitivity and responsiveness are usually determined by evaluating the relationship between changes in clinical and patient-rated endpoints and changes in the instrument over time, usually within in a clinical trial or observational study [29, 30].

So-called ‘distributional’ approaches are widely used to evaluate sensitivity to change and are based on the statistical features of the data produced by the instrument [31]. The simplest approach to assess change in health status is to calculate an ‘effect size’ which relates data on change produced by the instrument to variance, usually in the baseline data of the instrument [32]. A potentially superior approach, however, is to calculate the standard error of measurement (SEM) [33–35], which reflects the instrument’s reliability as well as its variance [36]. The SEM estimates the extent to which the observed change is a true change rather than measurement error; thus, any change score above the SEM can be considered statistically significant change in the sense that it is unlikely to have arisen by chance.

One limitation of SEM, however, is that it is based on information about scores at a single time point only rather than multiple time points. A more accurate measure of change would therefore require the calculation of the difference (S_diff_) between the SEM at two time points in a longitudinal study [33, 37]. We employed both approaches to assess sensitivity to change of the OxCAP-MH in this study.

### Aims

The aim of this study was to establish the psychometric properties of the OxCAP-MH in terms of internal consistency, test-retest reliability, convergent and discriminant validity and sensitivity to change in a clinical sample with psychotic illnesses.

## Methods

### Participants and setting

Data were collected at baseline as part of the Oxford Community Treatment Order Evaluation Trial (OCTET, trial registration number: ISRCTN73110773) between 2008 and 2012 [38]. Inclusion criteria were: aged 18-65 years, primary diagnosis of psychotic illness, currently detained for inpatient treatment, considered suitable for a Community Treatment Order (CTO, a legal regime mandating patients to adhere to treatment while living in the community), and able to give informed consent. Following recruitment, patients were randomised to leave hospital either on a CTO or to voluntary treatment and followed up for 12 months. The study was granted ethical approval by the Staffordshire NHS Research Ethics Committee [REC ref. 08/H1204/131] and all patients gave informed consent prior to interview.

### Study design

The reliability and validity analyses employed a cross-sectional design. The sensitivity to change analysis used a longitudinal design. All patients were interviewed at baseline and 12 months by trained researchers who administered the instruments below. Socio-demographic and clinical details were collected from medical records. Patients were identified via participating clinicians. Interviews for the OCTET study lasted approximately one hour (the OxCAP-MH took around five minutes to complete) and patients were reimbursed with £25 for each interview.

Test-retest data were collected as part of a follow-up of the OCTET study 48 months after randomisation. All participating patients were contacted twice with the same postal questionnaire with a seven-day interval between questionnaires [39]. To eliminate bias due to changes in patients’ mental state or social situation, patients were asked the following question in the second questionnaire: *Since you last completed this questionnaire, has anything in relation to your mental health or social situation changed?* Patients who answered ‘yes’ to this question were excluded from the test-retest analysis.

### Instruments

#### Capabilities

The OxCAP-MH is a patient reported outcome measure developed for use in mental health research. It was developed in several stages (see Simon et al. [26]). Initial testing of a longer (18-item) version led to the removal of two items (home ownership and life expectancy) following factor analysis. In a second version, two items (*Does your health affect your daily activities compared to most people your age?* and *Are you able to meet socially with friends and relatives?*) were dichotomously coded (yes/no) and then converted into a 1 to 5 scale (1 = 1 and 2 = 5) for scoring purposes, while all other items were scored on a 1 to 5 Likert scale (e.g. strongly agree, agree, neither agree nor disagree, disagree, strongly disagree). In the final application of the instrument, including the test-retest reliability, all 16 items were scored on 1 to 5 Likert scales.

The OxCAP-MH is scored on a 0–100 scale with higher scores indicating better capabilities. Scores are converted using the formula: 100 × (OxCAP-MH total score – minimum score)/range. Items 2, 4, 5, 6, 9, 10, 11, 12, 13, 14, 15 and 16 are reversed coded. A full version of the questionnaire can be found online at http://healtheconomics.meduniwien.ac.at/science-research/oxcap-mh/.

#### Health-related quality of life

*The EQ-5D* [11, 40] is a self-complete questionnaire that assesses health-related quality of life at the time of interview, and has two components. The EQ-5D-3L is a five-item questionnaire with three levels for each question, ranging from not present (1) to severe disability (3). Scores for the 3L are then converted to standardised ‘utilities’ based on UK population norms, ranging from −0.59 to 1, with 1 being the equivalent of perfect health and zero the equivalent of dead. The EQ-5D Visual Analogue Scale (VAS) is a 0 to 100 measure of current health status where 0 and 100 represent the worst and best imaginable health states respectively. The EQ-5D is a generic, multi-attribute instrument widely used in health economics research as the main outcome measure for cost-utility analyses. Reference to the EQ-5D in this study is to the 3 Level (3L) version throughout.

#### Psychopathology

*The Brief Psychiatric Rating Scale (BPRS)* [41, 42] is a clinician rated measure of psychiatric symptom severity based on the two weeks prior to interview. The instrument has 24 items that are rated on a seven point scale from not present (1) to extremely severe (7). It has a minimum score of 24 and maximum of 168, with higher scores indicating poorer functioning.

#### Overall functioning

*The Global Assessment of Functioning (GAF)* [43] is a clinician rated measure of overall functioning. It combines symptoms and social/occupational functioning into a single score from 0 to 100 with higher scores indicating superior functioning.

#### Objective social outcomes

*The Objective Social Outcomes Index (SIX)* [44] is a brief index used for benchmarking social outcomes by capturing objective information about an individual’s social situation in three domains: employment, living situation and social contacts. The instrument scores from 0 to 6 with higher scores indicating better outcomes.

### Statistical analyses

Data were checked for normality using the one-sample Kolmogorov-Smirnov test of goodness of fit. Descriptive statistics of socio-demographic and clinical characteristics used means (SD) for normally distributed data, medians (IQR) for non-normally distributed data, and number (%) for categorical data. Between-group comparisons used t-tests for normally distributed data, Mann-Whitney U tests for non-normal data, and Chi-square tests for categorical data.

Floor and ceiling effects on individual items in the index were calculated for all Likert scale items and considered present if more than 40% of patients scored the lowest or highest score respectively [28]; for total OxCAP-MH scores, effects were considered present if more than 15% of respondents scored the lowest or highest possible score respectively [45].

The reliability of the OxCAP-MH was evaluated in the following ways. The internal consistency was assessed using Cronbach’s alpha where values of 0.70 and over were considered satisfactory. Corrected item-total correlations were calculated to assess redundancy of individual items, with scores from 0.2 to 0.8 considered acceptable [28]. The test-retest reliability was established by calculating the intraclass correlation coefficient using a two-way random model with absolute agreement.

Convergent validity was assessed by calculating the Pearson correlation coefficients for the OxCAP-MH and the EQ-5D using UK tariff and VAS scores, while discriminant validity was determined by calculating the Pearson correlation coefficients for the OxCAP-MH and the BPRS, GAF and SIX with lower correlation values expected.

Sensitivity to change was assessed as follows. Baseline mean (SD), 12-month follow-up mean (SD) and internal consistency reliability coefficients were calculated for the OxCAP-MH (16-items). The baseline and follow-up SEM was calculated using the following formula:

$$ SEM={\sigma}_x\ \sqrt{1-{r}_{xx}} $$

Where σ is the standard deviation of the score and r is the reliability of the instrument. SEM scores were calculated from the baseline and 12-month follow-up OxCAP-MH scores. SEMs were then used to obtain the S_diff_ using the following formula:

$$ {S}_{diff}=\sqrt{\left({SEM}_1^2+{SEM}_2^2\right)} $$

There is currently no consensus about how many SEMs a score must change for it to be considered a clinically meaningful change for the individual. It has been argued that a difference of one-SEM frequently corresponds to a minimally important difference [35], although a more conservative approach can be used which multiplies the SEM by 1.96 and corresponds with the 95% confidence interval [46, 47]. Using higher SEM multiplier simply means that higher change scores are required to identify change scores that are unlikely to have arisen by chance. We report both.

All data were analysed using SPSS version 20 [48].

## Results

### Participant characteristics

A total of 336 patients were randomised in the OCTET Trial. Of these, one patients withdrew and two were ineligible [38]. Complete OxCAP-MH and other relevant outcome baseline data were available for 172 patients. Statistical analyses for the OxCAP-MH validation study were carried out on this sample. The characteristics of patients in this subsample did not differ significantly from the full cohort, other than there being more patients living homeless in the full cohort 35 (11%), compared to 3 (2%) in the sub-sample (Table 1).

**Table 1:** Socio-demographic and clinical characteristics of psychosis patients included in the OCTET RCT and patients with complete OxCAP-MH data (*N* = 172)

	OCTET RCT missing data (*N* = 333)	OCTET RCT (*N* = 333)	OxCAP-MH validation study (*N* = 172)
Age	0 (0%)	39.6 (11.4)	38.2 (11.2)
Sex	0 (0%)		
Male		224 (67.3%)	127 (72%)
Female		109 (33%)	48 (28%)
Years of education	4 (1.2%)	11.9 (1.9)	11.9 (1.9)
Ethnic origin	0 (0%)		
White British		204 (61%)	101 (59%)
Black		77 (27%)	43 (25%)
Asian		29 (9%)	16 (9%)
Mixed and other		23 (7%)	12 (7%)
Born in the UK	1 (<1%)	256 (77%)	137 (80%)
Marital status	2 (<1%)		
Single		247 (74%)	128 (74%)
Married/co-habiting		29 (9%)	19 (11%)
Separated/divorced		55 (17%)	25 (15%)
Have children	2 (<1%)	134 (40%)	67 (39%)
Living situation	2 (<1%)		
Independent accommodation		238 (72%)	142 (83%)
Supported accommodation		58 (17%)	27 (16%)
Homeless		35 (11%)*	3 (2%)*
Employment	1 (<1%)		
Incapacity benefit		292 (88%)	147 (85%)
Regular paid		2 (<1%)	2 (2%)
Voluntary/protected/sheltered		2 (<1%)	2 (2%)
Job seeker’s allowance		14 (4%)	9 (5%)
Unemployed, no benefits		13 (4%)	7 (4%)
Other (student/pensioner)		9 (3%)	5 (3%)
Clinical diagnosis	0 (0%)		
Schizophrenia		283 (85%)	153 (89%)
Other psychoses		50 (15%)	19 (11%)
BPRS	22 (7%)	38.7 (11.4)	37.3 (10.4)
GAF	24 (7%)	38.7 (9.7)	40.5 (9.6)
Duration of illness (years)	8 (2%)	14.3 (10.3)	13.2 (10.1)
No. of past psychiatric hospital admissions	21 (6%)	5 [3–9]	5 [3–8]

Data are number (%), mean (SD), or median [IQR]. *BPRS* Brief Psychiatric Rating Scale, *GAF* Global Assessment of Functioning

Percentages may not sum to 100 due to rounding

*Significant at the .05 level

Of the 172 patients included in the analysis, 124 (72%) were male; 101 (59%) were white, 43 (25%) were black, 16 (9%) were Asian and 12 (7%) were of ‘other’ ethnic origin; 153 (89%) had a primary diagnosis of schizophrenia, schizotypal or delusional disorder, while 19 (11%) had a diagnosis of other psychotic disorders (including bipolar disorder). Patients had a mean age of 38 years (SD = 11) and a mean illness duration of 13 years (SD = 10). At baseline, 147 (85%) of patients were receiving incapacity benefit; 142 (83%) had independent accommodation; 2 (2%) were in regular paid employment; and 19 (11%) were married or lived with a partner. Details of the socio-demographic and clinical characteristics of the participants are presented in Table 1.

### Floor and ceiling effects

A ceiling effect was identified in two items with 42% of respondents reporting having ‘very suitable’ accommodation and 43% reporting feeling ‘very safe’ walking alone near their home; a floor effect was observed in one item with 43% of respondents reporting ‘never’ losing sleep over worry in the past four weeks. Overall, however, there was no evidence of floor or ceiling effects in the total OxCAP-MH scores, with less than 15% of respondents scoring either the highest or lowest scores [45]. (Additional file 1).

### Reliability of the OxCAP-MH

The OxCAP-MH was found to have substantial internal consistency with Cronbach’s alpha of 0.79. Corrected item-total correlations were considered satisfactory and ranged from 0.20 to 0.59 [28]. Of the 311 test-retest reliability questionnaires sent out, 57 were completed at both time points and returned. Patients who returned the questionnaire were more likely to have independent accommodation compared to the overall sample contacted (86% vs. 71%, *p* < .05), but otherwise did not significantly differ in their baseline socio-demographic characteristics. Five patients who reported a change in their mental health or social situation were excluded from analysis. Two questionnaires were excluded due to missing data. A sample of 50 was retained for analysis.

**Fig. 1:**
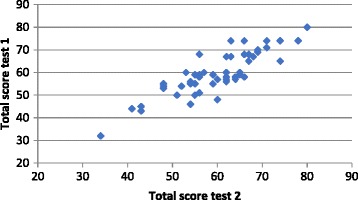
OxCAP-MH test-retest reliability based on total scores collected one week apart (*n* = 50)

The test-retest reliability analysis generated a single-measure intraclass correlation coefficient of 0.86 (*p* < .001) (Fig. 1). Linear regression produced a standardised coefficient of 0.86 (*P* < .001) and adjusted *R* ^*2*^ of 0.73, supporting the substantial reliability observed.

### Validity of the OxCAP-MH

Pearson correlations between OxCAP-MH total scores and the other instruments are presented in Table 2. Correlations were highest with the EQ-5D VAS (.522, *p* < .001, *n* = 171) and EQ-5D-3L Utilities (.452, *p* < .001, *n* = 170) followed by the BPRS (−.413, *p* < .001, *n* = 172). The negative association with the BPRS was expected as higher scores on this instrument indicate poorer functioning. A weaker association was observed between OxCAP-MH total scores and the GAF (.240, *p* < .001, *n* = 171) and SIX (.118, *p* = ns, *n* = 172). Correlations between the individual items of the OxCAP-MH and established measures of symptom severity, functioning, and outcome can be seen in Additional file 2.

**Table 2:** Pearson correlations between the OxCAP-MH total scores and established measures of illness severity, functioning and social outcomes

	OxCAP-MH	EQ-5D 3L	EQ-5D VAS	BPRS	GAF
EQ-5D-3L Utility	.452**				
EQ-5D VAS	.522**	.517**			
BPRS	−.413**	−.411**	−.325**		
GAF	.240**	.233**	.203**	−.443**	
SIX	.118	.129	.100	−.056	.159*

*EQ-5D 3L* EuroQol-3L Utility, *EQ-5D VAS* EuroQol Visual Analogue Scale, *BPRS* Brief Psychiatric Rating Scale, *GAF* Global Assessment of Functioning, *SIX* Objective Social Outcomes Index

** Significant at the .001 level * Significant at the .05 level

### Sensitivity to change

Complete data for the OxCAP-MH were available for 104 patients at both baseline and 12-months follow-up. The SEM values for baseline and follow-up and values for S_diff_ using the two criteria (one-SEM and 1.96*SEM) are presented in Table 3. Between baseline and follow-up there was a small increase in mean capability scores from 68 to 71.

**Table 3:** SEM values for the OxCAP-MH at baseline and 12-months follow-up

	Baseline (T1)		Follow-up (T2)		SEM Values					
	*N* = 104		*N* = 104		1 * SEM			1.96 * SEM		
	Mean (SD)	Alpha^a^	Mean (SD)	Alpha^a^	T1	T2	Diff.^2^	T1	T2	Diff.^b^
OxCAP-MH	67.67 (13.80)	0.78	70.81 (11.85)	0.70	6.47	6.49	9.16	12.68	12.72	17.96

*SEM* Standard error of measurement

^a^Cronbach’s alpha coefficient

^b^ $$ {S}_{diff}=\surd \left({SEM}_1^2+{SEM}_2^2\right) $$

Using the one-SEM of change criterion, a score of 6.47 on a 0–100 scale can be considered a statistically important difference. This cut off increases to 12.68 when the more conservative 1.96 * SEM criterion is applied. The standard error of the difference (S_diff_) shows that a minimally significant change from baseline to 12-months follow-up corresponds to a 9.16 points of change on a 0–100 scale; this threshold increases to 17.96 when the 1.96*SEM criterion is used.

Using the one-SEM S_diff_ criterion of statistically significant change between baseline and follow-up (9.16), 24 (23%) patients improved, 67 (64%) showed no change, and 13 (12%) deteriorated. For these three groups, the mean (SD) capabilities scores at 12-months follow-up were 74.5 (11.5), 70.0 (12.3) and 68.4 (8.8). Using the more stringent 1.96*SEM S_diff_ threshold of 17.96, 8 (8%) patients improved, 92 (88%) showed no change, and 4 (4%) deteriorated. These three groups had mean (SD) capabilities scores at 12-months follow-up of 74.8 (12.2), 70.7 (12.0) and 65.2 (5.6) respectively.

### Distribution of the OxCAP-MH scores

**Fig. 2:**
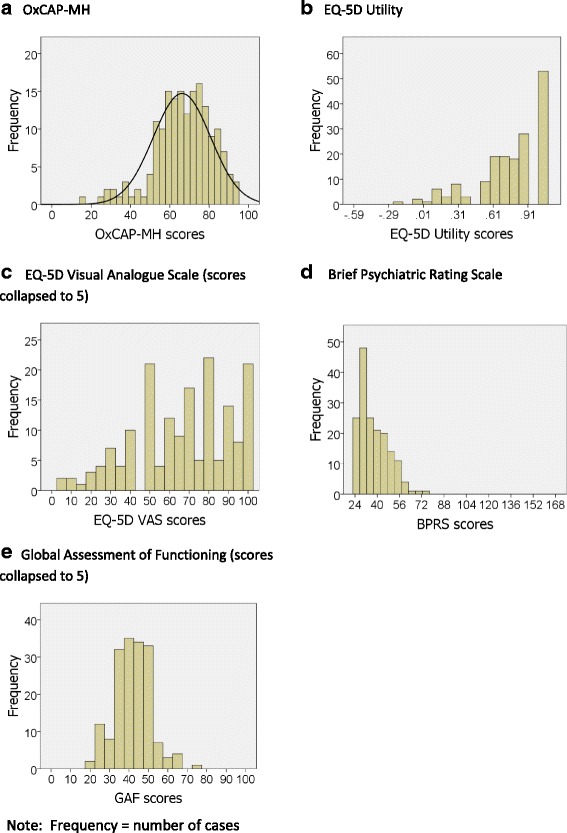
Distribution of patients’ total scores for instruments used in psychometric validation (panels **a** – **e**). Frequency = number of cases

The distribution of total scores for the OxCAP-MH, EQ-5D-3L Utilities, EQ-5D VAS, BPRS and GAF are presented in Fig. 2 (Frequency = number of cases). Panel A shows that patients’ total scores for the OxCAP-MH are normally distributed.

## Discussion

This study reports the statistical evaluation of the psychometric properties of a multi-dimensional capability instrument designed for use in a mental health context. The instrument showed strong psychometric properties.

### Reliability and validity

The OxCAP-MH was found to have good reliability: the internal consistency was evidenced by a Cronbach’s alpha of 0.79 whilst the test-retest reliability measured by an intraclass correlation coefficient of 0.86 – both substantial correlations significant at the *p* < 0.001 level. Results also support the validity of the OxCAP-MH. The convergent validity of the instrument was demonstrated by its strong correlation with established measures of health-related quality of life (EQ-5D) and illness severity (BPRS). The modest correlation with overall functioning (GAF) and an objective measure of social outcomes (SIX) supports the divergent validity of the instrument. The strength of these associations can be partially accounted for by the theoretical relationship between the instruments.

The EQ-5D is a widely used generic measure of health-related quality of life. It has undergone extensive reliability and validity testing with a range of health conditions [49–54] and has arguably the closest theoretical association with the OxCAP-MH. The EQ-5D and OxCAP-MH both capture patients’ subjective appraisal of their own quality of life evidenced by the strong and statistically significant correlation between the instruments. A perfect correlation between the instruments would not be expected, however, since the OxCAP-MH is designed to capture a much wider range of outcomes than the EQ-5D including health and non-health domains. The OxCAP-MH should capture health-related quality of life and well-being but, as a multi-dimensional measure, it should also capture *more*. Interestingly, the OxCAP-MH correlated more strongly with the EQ-5D VAS scores than with the Utility scores. One possible explanation for this is that the Utility scores capture specific aspects of quality of life – namely those that are health-related – while the VAS reflects the patient’s judgement about their overall health status, which is arguably more in line with the aims of the OxCAP-MH which attempts to capture the patient’s overall well-being [26]. Furthermore, because the dimensions of life quality measured in the OxCAP-MH are conceptually diverse, the moderately high Cronbach alpha suggests that the severe mental illnesses examined have significant impact on most aspects of quality of life.

In contrast to the EQ-5D, correlations with the GAF – a well-established clinician-rated measure of overall (clinical and social) functioning – was 0.24, while the association with the SIX – an objective index of social outcomes – was just 0.12. Again, these associations can be explained by the more distal theoretical relationship between the GAF and SIX and the OxCAP-MH. The GAF score represents a patient’s overall functioning as perceived by the clinician/researcher, while the SIX merely captures objective ‘facts’ about their social situation (like having employment) – neither would be expected to correlate highly with a patient’s subjective appraisal of what they feel free to be and do i.e. their capabilities. The validity of the OxCAP-MH is further supported by its significant negative association with the BPRS. This indicates that there is a strong negative relationship between patients’ capabilities and psychopathological symptoms. Associations between all instruments used in this study and the 16 individual items of the OxCAP-MH are presented in Additional file 2.

Ceiling effects were observed in two items in the OxCAP-MH with 42% of respondents reporting having ‘very suitable’ accommodation and 43% reporting feeling ‘very safe’ walking alone near their home. These response rates may reflect the particular wording of the questions and the fact that the respondents live in a wealthy industrialised country in which the majority of people do have suitable accommodation and are subject to relative low levels of crime. A floor effect was observed in one item with 43% of respondents reporting ‘never’ losing sleep over worry in the past four weeks. Although a small number of items demonstrated floor and ceiling effects they were retained in the measure as they were regarded as important and contributed to the content validity of the instrument. Furthermore, overall domain scores did not indicate any such floor/ceiling effects.

### Sensitivity to change

The results show that on average there was little change in mean capabilities scores between baseline and 12-months follow-up, a finding that is consistent with results from the OCTET Trial including secondary and follow-up analyses [38, 55, 56]. The results show that using the one-SEM criterion, a change of around 9.2 on the OxCAP-MH 0–100 scale is unlikely to be due to measurement error and can be considered on distributional grounds to be a true change in score. When the more conservative 1.96 * SEM criterion is applied, this threshold for true change increases to 18.0 points of change. It is important to remember that these changes are not necessarily clinically meaningful but rather represent differences that, on statistical grounds, are unlikely to have arisen by chance.

The inclusion of approximate 95% confidence intervals (1.96 * SEM or 1.96 * S_diff_) substantially increased the minimum significant – or ‘real’ – change score required for the OxCAP-MH. Fitzpatrick and colleagues [33] note that ‘for group-based evaluative research there is a risk that calculating minimum change scores by distributional methods adjusted for 95% confidence intervals will result in too conservative an approach with respondents who experience important deterioration being missed and treated as unchanged’ (p.1413). The use of one-SEM or one-S_diff_ would be in keeping with methods used in several studies assessing change across a range of health-related quality of life instruments in patients with asthma, cardiac problems, Parkinson’s disease, amyotrophic lateral sclerosis, and chronic obstructive pulmonary disease [33–36, 57]. In these studies, one-SEM was considered the optimal statistical criterion consistent with anchor-based evidence [36]. Using the one-SEM S_diff_ criterion, an individual change score between baseline and 12-months follow-up that is greater than 9 points would be considered an improvement or deterioration in capabilities scores that did not arise due to chance or measurement error.

The question of whether to use one-SEM (or one-S_diff_) or to use confidence intervals and therefore more stringent criteria for minimal change partly depends on whether decisions are made with respect to groups or individuals – for example, interpretation of clinical trials, or an individual patient in a clinical context [36]. In general, the group context is associated with greater confidence in any given estimate of health related quality of life while a clinician making health-related quality of life decisions at the individual level may opt for the more conservative approach gained from the confidence interval adjusted scores to determine change [33, 36].

### Distributions of OxCAP-MH scores

Figure 2 shows that compared with other indicators of functioning and outcome used in this study, the OxCAP-MH total scores follow a more normal distribution. This is important because an instrument that is normally distributed is less likely to lose information to floor or ceiling effects, which can compromise validity. For example, a well-known limitation of the EQ-5D is its propensity for ceiling effects, a finding that has been shown repeatedly in range of patients groups [9] and the general population [58]. Problems of sub-optimal score distribution have also been observed for the GAF. Reliability studies show that GAF scores can have restricted distributions and can be unreliable; in one study 20% of raters accounted for more than 50% of the spread of scores, and deviations can be 20 points or more [59–61]. Figure 2 shows that the distribution of OxCAP-MH total scores are not affected by floor or ceiling effects.

### Strengths and limitations

The majority of concepts used to assess quality of life have been introduced into healthcare not on the basis of a theoretical model but rather on the basis of convenience or intuitive appeal [62]. A major strength of the capability approach is therefore its theoretical pedigree. Sen’s work proposes a model of human welfare that is based on substantive freedoms to achieve the things that an individual has reason to value rather than on relying on resource and desire fulfilment typical of many traditional quality of life frameworks [22]. Moreover, the OxCAP-MH focuses on factors that link directly to peoples’ broader well-being rather than relying on proxies (such as health-related quality of life) as is the case with the EQ-5D.

Measuring capabilities remains a challenge, however, and there is ongoing theoretical discussion about which capability domains are most important and how they ought to be measured [18, 63]. The capability approach is not directly linked with traditional conceptual frameworks of health and quality of life, and there are comparatively few capability measures with which to compare new instruments. The development and validation of a novel capability measure represents an important conceptual and methodological development within the capabilities literature as well as research on health measurement.

The data used in this study could not fully address the question of sensitivity to change of the OxCAP-MH. In particular, the absence of a patient-rated anchor question at 12-months follow-up means that the clinical meaningfulness of the instrument could not be tested. Among the existing capability measures developed for use in health contexts, evidence supporting their sensitivity to change is limited and somewhat mixed [64–66]. Coast and colleagues [18] note that generic capability measures cover a very broad informational space – i.e. the entirety of the individual’s life rather than just their health for example – which may make it more difficult to demonstrate their sensitivity to change. These challenges notwithstanding, demonstrating the sensitivity to change of the OxCAP-MH remains essential if the instrument is to be useful for distinguishing different interventions and should be tackled in future studies.

Patients in this sample were mostly out-patients with severe mental illnesses and further work is needed to establish the feasibility and validity of using the instrument in other settings (e.g. in-patient care) and with other patient groups. Finally, it is also worth noting that male participants were slightly over represented in this sample and equal representation should be considered in future studies.

## Conclusions

The statistical validation described above shows that the OxCAP-MH, the first mental health specific capabilities instrument has good psychometric properties in terms of reliability and validity. Some questions about the instrument’s sensitivity to change remain, however, and further work with larger samples that include explicit anchor-based questions is necessary. The results support the use of self-reported capabilities to assess outcomes in patients with severe mental illness for clinical, health services and economic evaluations. The OxCAP-MH is now freely available for non-profit purposes at: https://healtheconomics.meduniwien.ac.at/science-research/oxcap-mh/.

## Additional files

Additional file 1: Floor and ceiling effects for OxCAP-MH items scored on a 1 to 5 Likert scale. (DOCX 13 kb)

Additional file 2: Correlation of individual items of the OxCAP-MH with established measures of illness severity, functioning and social outcomes. (DOCX 13 kb)
