# Supplementary material for: Psychometric validation of a multi-dimensional capability instrument for outcome measurement in mental health research (OxCAP-MH)
Source: Health Qual Life Outcomes. 2017 Dec 28;15:250. doi: 10.1186/s12955-017-0825-3 (PMC5745777; doi:10.1186/s12955-017-0825-3)
Supplement: Supplementary file 1 — Floor and ceiling effects for OxCAP-MH items scored on a 1 to 5 Likert scale. (DOCX 13 kb) [file 12955_2017_825_MOESM1_ESM.docx]

## Additional file 1: Floor and ceiling effects for OxCAP-MH items scored on a 1 to 5 Likert scale

| **Item** | **Floor** | **Ceiling** |
| --- | --- | --- |
| 1. Does your health limit your daily activities | n/a | n/a |
| 2. Able to meet socially with friends and relatives | n/a | n/a |
| 3. In past 4 weeks, how often lost sleep over worry | 43%* | 2% |
| 4. In the past 4 weeks, how often able to enjoy recreational activities | 10% | 12% |
| 5. Suitable / Unsuitable accommodation for current needs | 7% | 42%* |
| 6. How safe feel walking alone near home | 5% | 43%* |
| 7. Able to influence decisions affecting local area | 6% | 11% |
| 8. Free to express views | 10% | 4% |
| 9. Able to appreciate and value plants, animals and world of nature | 28% | 1% |
| 10. Respect, value and appreciate people around me | 26% | 1% |
| 11. I find it easy to enjoy the love, care and support of my family | 27% | 4% |
| 12. Free to decide how to live life | 12% | 6% |
| 13. Free to use imagination and express myself creatively | 14% | 4% |
| 14. Have access to interesting forms of activity or employment | 11% | 5% |
| 15. How likely will be assaulted in future | 39% | 6% |
| 16. How likely will experience discrimination | 31% | 12% |

* Denotes floor / ceiling effect (>40%); n/a = non-applicable (these items were scored yes/no responses in the previous version of the instrument)
