# Supplementary material for: Psychometric validation of a multi-dimensional capability instrument for outcome measurement in mental health research (OxCAP-MH)
Source: Health Qual Life Outcomes. 2017 Dec 28;15:250. doi: 10.1186/s12955-017-0825-3 (PMC5745777; doi:10.1186/s12955-017-0825-3)
Supplement: Supplementary file 2 — Correlation of individual items of the OxCAP-MH with established measures of illness severity, functioning and social outcomes. (DOCX 13 kb) [file 12955_2017_825_MOESM2_ESM.docx]

## **Additional file 2:** Correlation of individuals items of the OxCAP-MH with established measures of illness severity, functioning and social outcomes

|  | OxCAP-MH individual items† | | | | | | | |
| --- | --- | --- | --- | --- | --- | --- | --- | --- |
|  | *Item 1* | *Item 2* | *Item 3* | *Item 4* | *Item 5* | *Item 6* | *Item 7* | *Item 8* |
| EQ-5D 3L | .457** | .270** | .346** | .197** | .109 | .206** | .080 | .101 |
| EQ-5D VAS | .473** | .320** | .183* | .225** | .236** | .167* | .076 | .274** |
| BPRS | -.338** | -.170* | -.453** | -.173* | -.238** | -.226** | -.043 | -.158* |
| GAF | .266** | .092 | .131 | .109 | .013 | .148 | .069 | .044 |
| SIX | -.016 | .170* | .057 | .008 | -.015 | .050 | .054 | .055 |
|  |  |  |  |  |  |  |  |  |
|  | *Item 9* | *Item 10* | *Item 11* | *Item 12* | *Item 13* | *Item 14* | *Item 15* | *Item 16* |
| EQ-5D 3L | .023 | .031 | .215** | .243** | .264** | .199** | .271** | .269** |
| EQ-5D VAS | .124 | .082 | .307** | .286** | .330** | .287** | .272** | .277** |
| BPRS | -.064 | .031 | -.228** | -.161* | -.254** | -.173* | -.231** | -.248** |
| GAF | .078 | .017 | .273** | .137 | .073 | .163* | .089 | .076 |
| SIX | .147 | .173* | .150 | .029 | .006 | .113 | .046 | .018 |

*EQ-5D 3L = EuroQol 3 level; EQ-5D VAS = EuroQol Visual Analogue Scale; BPRS = Brief Psychiatric Rating Scale; GAF = Global Assessment of Functioning; SIX = Objective Social Outcomes Index*

*** Significant at the .001 level * Significant at the .05 level*
